# Tumor−associated macrophage polarization in the inflammatory tumor microenvironment

**DOI:** 10.3389/fonc.2023.1103149

**Published:** 2023-02-02

**Authors:** Zijuan Zou, Hongfen Lin, Mengsen Li, Bo Lin

**Affiliations:** ^1^ Hainan Provincial Key Laboratory of Carcinogenesis and Intervention, Hainan Medical College, Haikou, Hainan, China; ^2^ Institution of Tumor, Hainan Medical College, Haikou, Hainan, China

**Keywords:** tumor-associated macrophages, polarization, inflammatory, tumor microenvironment, cancer immunotherapy of macrophages

## Abstract

The chronic inflammation of tumor continues to recruit TAMs (tumor−associated macrophages) to the TME (tumor microenvironment) and promote polarization. Pro-inflammatory signals polarize macrophages to the M1 phenotype to enhance inflammation against pathogens. Tumor inflammatory development changes the pro-inflammatory response to an anti-inflammatory response, resulting in the alteration of macrophages from M1 to M2 to promote tumor progression. Additionally, hypoxia activates HIF (hypoxia-inducible factors) in the TME, which reprograms macrophages to the M2 phenotype to support tumor development. Here, we discuss the factors that drive phenotypic changes in TAMs in the inflammatory TME, which will help in the development of cancer immunotherapy of macrophages.

## 1 Introduction

Macrophages are innate immune cells that play a key role in inflammation. At the initiation of inflammation, the number of neutrophils in the circulation increases, followed by monocytes that differentiate to macrophages to promote inflammation against invading pathogens (1). Further inflammation or chronic inflammation can result in tissue damage, and macrophages can also assist in preventing excess inflammation from occurring to protect the body (1). Macrophages in the inflammatory microenvironment eliminate invading pathogens, damaged tissue and apoptotic host cells, which further lead to the resolution of inflammation and tissue reparation (2). It was found that infected tissue without macrophages had an increased apoptotic neutrophil population and prolonged inflammation and tissue damage (3).

Because inflammation has the potential to cause harm, the inflammatory process is typically tightly regulated by macrophages. Pro-inflammatory or activity signals, such as interferon-γ (IFN-γ), colony-stimulating factor-1 (CSF-1), and lipopolysaccharide (LPS), polarize macrophages to the M1 phenotype to promote inflammatory development. Because non-resolving inflammation damages tissue, inflammation should be shut down by anti-inflammatory signals, such as IL-10 and transforming growth factor beta (TGF-β), that activate macrophages to resolve the inflammatory process (4). An abnormal regulation between pro-inflammatory and anti-inflammatory signals drives many diseases (4).

In the inflammatory TME, the proportion of macrophages can be as high as 30%-50%, and their function has been considered as the ‘soil’ for tumor growth. At the earliest stage of the tumor, macrophages polarize to M1 to generate an antitumor response. However, once tumors progress past the initial state, the macrophages polarize to M2 to promote tumor progression and malignancy (4–6). Tumors are also considered ‘wounds that do not heal’ that lead to chronic inflammatory and imbalanced polarization of macrophages (7–9). The present review provides an overview of macrophage polarization in inflammatory TME and proposes a therapeutic strategy for treating cancer.

## 2 Distribution of macrophages in the TME

**Figure 1:**
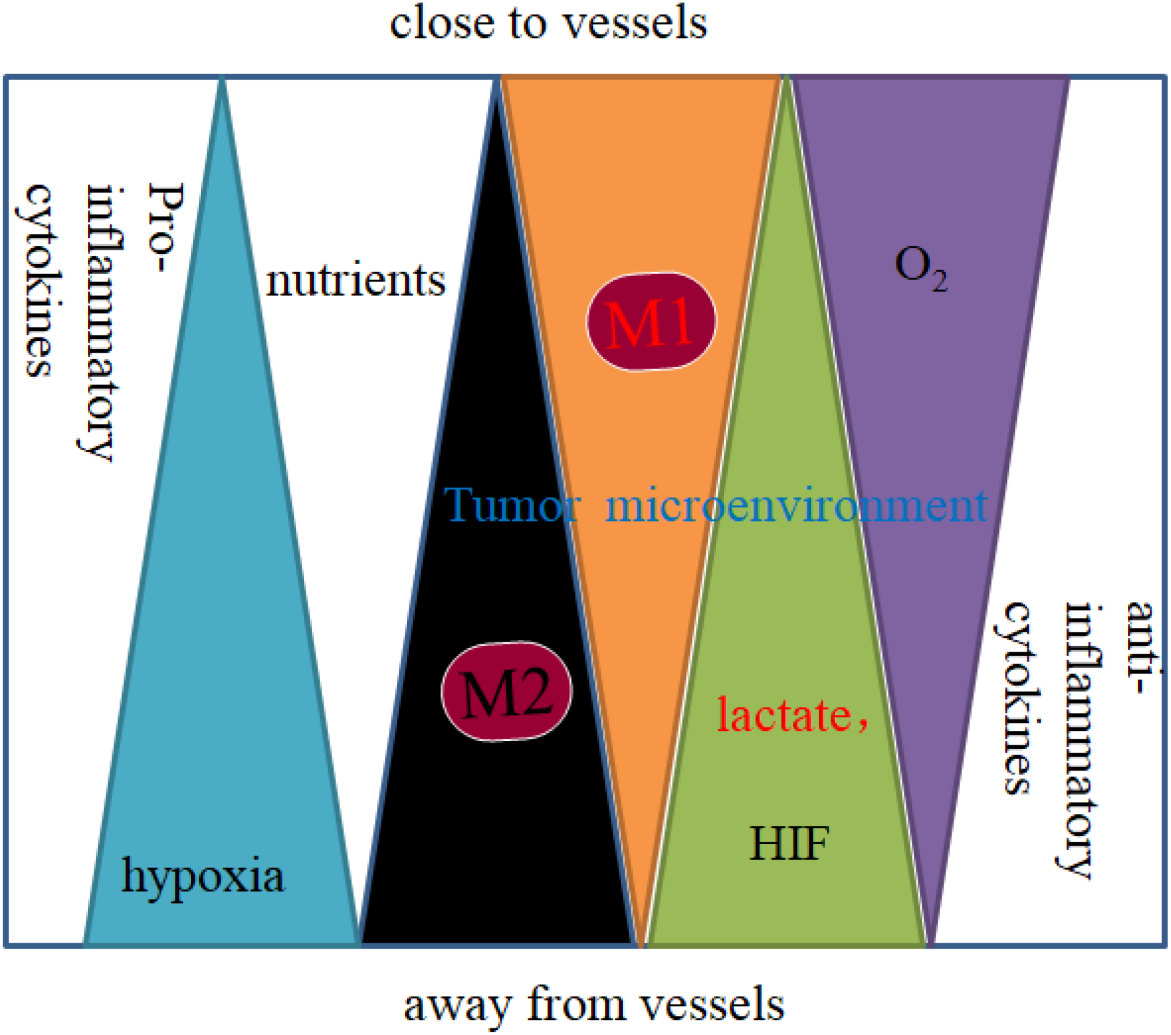
Distribution of macrophages in the tumor microenvironment.

Macrophages polarize to different phenotypes in response to signals and cytokines in their environment. Many factors affect TAM polarization and distribution, such as inflammatory signals and cytokines in the TME. The distribution of polarized TAMs in the tumor microenvironment is shown in **Figure 1** (9). As an important component of leukocytes, TAMs are mainly derived from circulating monocytes, tissue residue macrophages and myeloid-derived cells (MDSCs). Under specific conditional stimulation and an unequal distribution of nutrients created by the TME, macrophages can be polarized to the M1 type (classically activated phenotype, with markers such as CD80/86) and M2 type (alternatively activated phenotype, with markers such as CD206, CD163, CD204, and stabilin-1), which play an important role in carcinogenesis and metastasis (10). The unequal distribution of oxygen and nutrients in TME affects macrophage polarization. Macrophages are near perfused vessel areas, where nutrients such as glucose, glutamine, and oxygen are high, which induces macrophage polarization to the M1 type. Macrophages residing away from vessels in an environment of chronic hypoxia and a high concentration of lactate are induced to polarize to the M2 type (**Figure 1**) (1, 9). Both M1 and M2 macrophages are found in TME, whereupon cells in hypoxic areas show more dominant M2 activation. For example, TAMs can express both the M1 marker CD80 and the M2 marker CD206. However, in hypoxic areas, the M1 marker CD80 is expressed at lower levels, and the M2 marker CD206 is expressed at higher levels than in high oxygen areas (11).

Initiation of inflammation in the TME and pro-inflammatory cytokines, such as interleukin (IL)-1-β, IL-6, and IL-8, are produced by tumor cells, immune cells and nonmalignant cells that promote M1 polarization (12). IL-4, macrophage colony-stimulating factor (M-CSF), or granulocyte-macrophage colony-stimulating factor (GM-CSF), as well as IL-10, TGF-β and HIF-1a produced by the hypoxic TME, can skew macrophages to the M2 phenotype. For example, HIF-1a in hypoxic melanoma cells induced translocation and secretion of IL-10, which induced macrophage activation to the alternative M2 phenotype (13).

## 3 Factors affecting the polarization of tumor macrophages in TME

### 3.1 Inflammation

In TME, cells face hypoxia, nutrient deprivation and metabolic stress that cause sustained apoptosis and death. In the chronic inflammatory TME, apoptotic cells produce ‘find-me’ signals to recruit lymphocyte cells that produce inflammatory signals to keep out damaged tissue and prevent their own clearance (9), and the recruitment of neutrophils followed by monocytes that initiate inflammatory signals activates macrophages to the M1 phenotype (8).

But the chronic inflammation leads to the production of anti-inflammatory signals and the transition of macrophages from the M1 to anti-inflammatory M2 phenotype to prevent excess inflammation from occurring (14). M2 macrophages further secrete anti-inflammatory signals, such as IL-10 and TGF-β, to promote angiogenesis, remodeling, and immune suppression, which increase cancer cell proliferation, metastasis and resistance to therapy (15).

**Figure 2:**
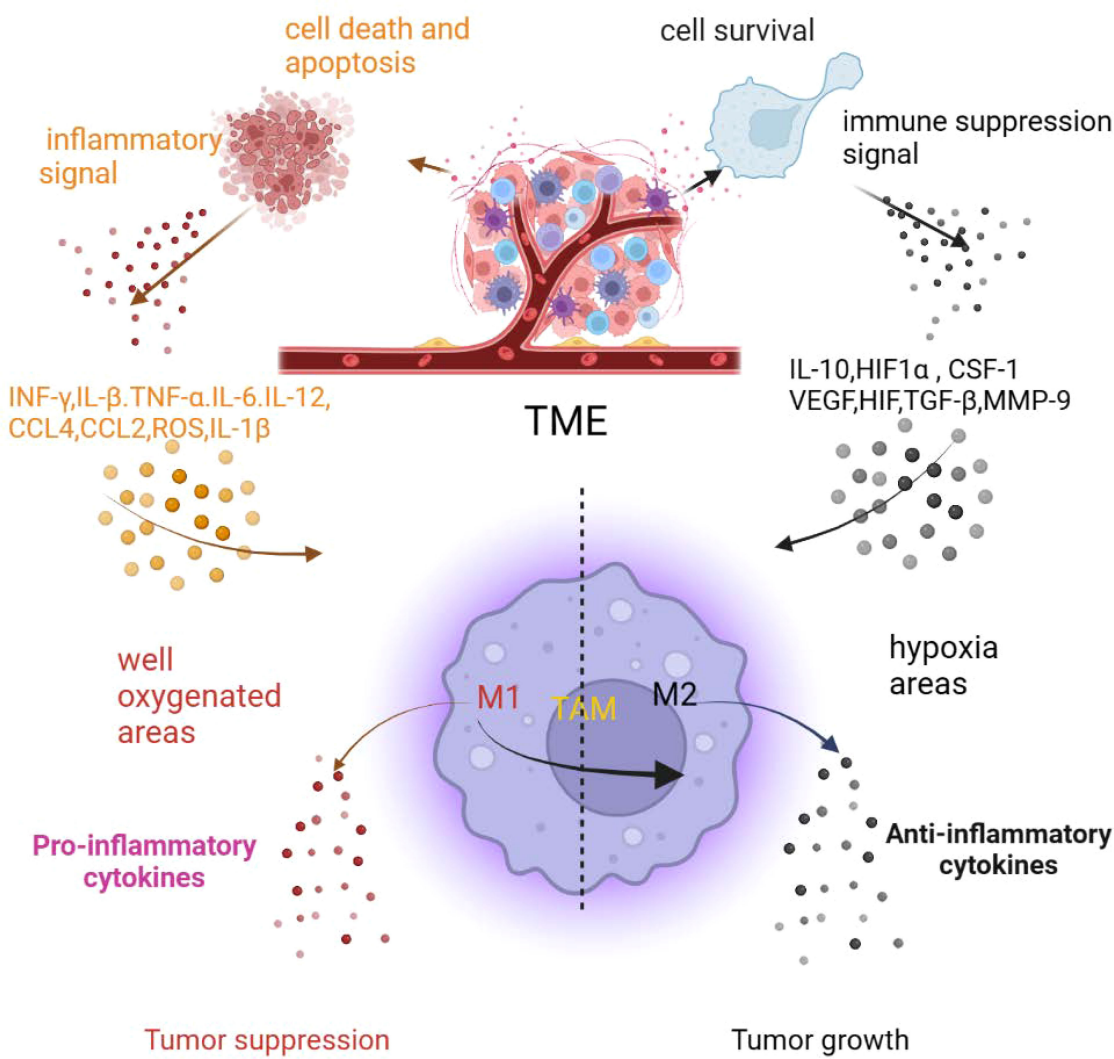
The inflammatory tumor microenvironment affects macrophage polarization (created in BioRender.com) (9).

Chronic cancer-associated inflammation also contributes to the TME producing cytokines and chemokines, such as IL-4, IL-6, IL-10, IFN-γ, CCL2, CCL5, CD40L, and TNF (8, 16–18). The cytokine and chemokine balance of pro- and anti-inflammatory mediators is a key factor in the progression of macrophage polarization and tumor development. An abundance of pro-inflammatory cytokines and chemokines in the TME recruit and polarize macrophages to the M1 phenotype and promote inflammation. With the development of inflammation, non-resolving cancer inflammation also produces an anti-inflammatory signal to inhibit inflammation that alters macrophages from the M1 to M2 type (9). As mentioned before, The chronic inflammation of tumors continue to cause pro-and anti-inflammatory response occurring, result in sustained polarization macrophages from the M1 to M2 type in the TME (**Figure 2**) (9).

In the clinic, macrophage polarization is strongly related to tumor stage; in the early phases of tumor inflammation, the TME recruits and polarizes more macrophages to the M1 phenotype, and in the tumor advanced state, more M2 macrophages are found, suggesting a dynamic switch from the M1 to the M2 phenotype (19). Moreover, several studies in murine and human tumors also observed a “mixed” macrophage phenotype in the TME, and the phenotype of macrophages also differs from tumor to tumor or within different areas of the same tumor (20, 21), and macrophages in an advanced state of tumors show a more dominant M2 marker expression pattern (11). Cytokines and chemokines secreted by pro- and anti-inflammatory signals can alter the physiological development of macrophages. It is known that at the earliest stage of the tumor, pro-inflammatory M1 macrophages are activated, and with tumor development, macrophages will convert to the immunosuppressive M2 phenotype in cancer nests to promote tumor growth (4–9, 15, 22, 23).

### 3.2 Hypoxia

The TME creates an unequal distribution of oxygen and nutrients that affect TAM polarization. In well oxygenated areas of the TME, macrophages show some qualities of classical (M1) activation. In contrast, in hypoxic areas, the TME produces HIF, TGF-β, or IL-6, which provokes alternative (M2) activation of macrophages to promote tumor progression.

Hypoxic stress in the TME not only alters the metabolism of macrophages but also alters their phenotype (9). Hypoxia activates HIF transcription factors to enhance HIF-dependent gene expression and promote the accumulation of the HIF-1/2 protein to adapt to oxygen shortage and metabolic stress. The pathways regulated by HIF can increase glycolysis and suppress O2 consumption. In hypoxic areas, nutrients also become scarce, and HIF enhances the Otto Warburg effect and alters metabolites to express more lactate and kynurenine to promote tumor cell proliferation. Due to the high concentrations of lactate, chemokines, and HIF-1/2 secreted from the hypoxic TME, macrophages are drawn to hypoxic areas and polarize to the M2 phenotype. HIF activation in the hypoxic TME also induces the expression of a number of genes, such as VEGF or matrix metalloprotease 9 (MMP9), that affect macrophage polarization and drive tumor progression (24). In the hypoxic TME of melanoma, tumor cells accumulate HIF-1 and also release high mobility group box 1(HMGB-1), which induces macrophages to produce IL-10 driving them to an M2-like phenotype that promotes proliferation and metastasis (13).

Hypoxic and nutrient stresses not only alter the phenotype of macrophages but also reprogram them. Hypoxic and nutrient stresses also provoke cell apoptosis, necroptosis, and autophagic death. To survive in oxygen- and nutrient-deprived TME, cells promote autophagy signaling pathways, but this promotion is always excessive and causes cell apoptosis and death. Apoptotic and dead cells are recognized by phagocytes that recruit and polarize macrophages to the TME. As mentioned before, the inflammation of tumors cause sustained cell apoptosis and death occurring in the TME, resulting in recruitment of macrophages and direct polarization of macrophages to the M1 phenotype; then, the hypoxic TME promotes the transition of macrophages from the M1 phenotype to the M2 phenotype or directly polarizes macrophages to the M2 phenotype to support tumor development (**Figure 2**) (9).

### 3.3 Tumor cells

In the TME, tumor cells recruit and reeducate macrophages to adopt a special phenotype by secreting vascular endothelial growth factor (VEGF), platelet-derived growth factor (PDGF), TGF-β, CCL2, or M-CSF (17, 25, 26). Hypoxic tumor cells also produce high amounts of lactate and HIF to polarize macrophages to the M2 phenotype to block effective antitumor immune responses by inhibiting tumor surveillance by T and NK cells (9, 27, 28). High lactate levels produced by tumor cells also evoke HIF-1a and HIF-2a accumulation in macrophages, which changes the pro-inflammatory environment to an anti-inflammatory environment by reducing NF-kB activity, in turn reducing T and NK-cell activation (9, 29). Tumor cells also promote membrane cholesterol efflux induces IL-4-mediated signaling in macrophages and alters their phenotype to promote tumor invasion and metastasis (30, 31). It was found that co-culturing macrophages with tumor cells increased HIF-1 and VEGF expression, which induced the dysregulation of arginase and Fizz1, and this was correlated with a gene signature found in alternatively activated macrophages that promote tumor development (9, 32).

The influence of tumor cells and macrophages is interactive. For example, when TAMs are cocultured with hepatoma cells, macrophage-derived IL-6 and IL-8 activate JAK kinase, which phosphorylates STAT3 activating STAT3 signaling in tumor cells and promotes the epithelial mesenchymal transition(EMT), thus enhancing tumor invasion and metastasis (33, 34). M2 TAMs can also induce high expression of both PD-L1 and CTLA4 in cancer cells, which promotes immune escape through limiting activationof cytotoxic T cells in the TME. TAMs induce high levels of PDL1 expression that correlate with poorer clinical outcomes in hepatocellular carcinoma (HCC) (8, 35–39).

### 3.4 Immune cells

Regulatory immune cells, such as Treg cells, MDSCs and B cells, can also regulate macrophage polarization. Treg cells inhibit CD8+ T-cells secreted IFN-γ, that maintain macrophages in the M2-like phenotype, which also reduces fatty acid oxidation and induces lipid accumulation in macrophages by increasing the expression of sterol regulatory element binding protein 1 (SREBP1) (40). Inflammatory interleukin-17-positive (IL-171) T cells can recruit and promote maturation of chemokine receptor 3–positive (CXCR3) B cells, which induce M2b macrophage polarization in human HCC (41). Tumor-infiltrating lymphocytic B cells program macrophages to the M2 phenotype *via* Bruton tyrosine kinase (BTK) activation in a PI3K-Y manner and inhibit B-cell infiltration. Inhibition of BTK in the pancreatic TME reduced tumor growth and enhanced antitumor activation (42).

MDSCs are heterogeneous immune cells that consist of myeloid progenitor cells and immature myeloid cells (IMCs). They can differentiate into TAMs and can affect macrophage polarization within TME. MDSCs can suppress the immune response by abnormally regulating STAT3 to promote anti-inflammatory (M2-like) macrophage polarization (31, 43). MDSCs are usually recruited to the TME and produce IL-10, which inhibits macrophage expression of IL-12 and alters the macrophage phenotype to M2. MDSCs also express high levels of arginase-1, which promote macrophage polarization and contribute to immune suppression (8, 43, 44).

### 3.5 Chemokines and cytokines

Macrophage polarization in the TME is dynamic and dependent on the balance of chemokines and cytokines. Numerous chemokines (such as CCL2, CCL5, CCL15, and CCL20) and cytokines (such as TGF−β, CSF-1 and TNF) have been demonstrated to participate in the mechanism of monocyte-derived macrophage recruitment, migration and polarization (45–49). The representative molecules are discussed below.

#### 3.5.1 CCL2

CCL2 is a small chemokine which is mainly produced by tumor cells and surrounding stromal cells. CCL2 recruit CCR2+ inflammatory monocytes from the bone marrow to the peripheral blood that lead to cancer metastases and poor clinical outcomes (50). CCL2 elevation in the TME is essential for the recruitment and education of monocyte-derived macrophage polarization. Macrophages express CCR2 were recruited by CCL2 that result in up regulating their expression levels of angiogenic factors, such as IL− 6, VEGF, and MMP9, which contributed to tumor vascularization. Inhibition of the CCL2/CCR2 signaling pathway can block monocyte recruitment and suppress the polarization of macrophages toward the M2 phenotype (51–53).

#### 3.5.2 CSF-1

Colony-stimulating factor 1 (CSF-1) involve in macrophage recruitment, differentiation, mature, and survival. CSF-1 receptor (CSF-1R) is a tyrosine kinase receptor which mainly expressed on monocytic lineages which will differentiate into TAMs. CSF-1 and IL-34 bind to CSF-1R active cascade of signaling in monocytes will increase recruitment of M2-like phenotype and promote immunosuppression (54). Tumor-derived CSF-1 promotes tumor growth and enhances M2 polarization and infiltration. Targeting CSF-1/CSF-1R signaling in combination with CXCR2 antagonists can prevent M2 polarization and shows a strong antitumor effect (55). It was found that CSF-1/CSF-1R signaling inhibition can reduce TAM infiltration and enhance the CD8+/CD4+ T-cell ratio to kill tumor cells. In a transgenic mouse model, targeting TAMs by CSF-1R blockade enhanced the anticancer efficacy of platinum-based chemotherapies (56, 57). It was also found that combination treatments of CSF-1/CSF-1R inhibitors with PD1-PDL1 inhibitors are promising candidates for effective elimination of TAMs (54).

#### 3.5.3 IL-6

IL-6 is an important cytokine, which is closely related to the malignant behavior, such as promotion of inflammation, proliferation, angiogenesis, invasion, metastasis of tumor in TME. It was found that IL-6 was a risk factor that highly expressed in chronic inflammatory tumor tissue that lead to poor prognosis (58). In inflammatory TME, IL-6 secreted by TAMs resulting in a vicious cycle that further promote macrophages polarization to TAMS and increase IL-6 expression which can lead to a smoldering inflammatory state, and enhance tumor cell metastasis (46, 59). Chen S,et al. showed that IL-6 was responsible for TAMs induced renal cell carcinoma cells migration, invasion, EMT by activating I L-6/STAT3 signaling (60). IL-6 acts on IL-6R/gp130 receptors and active STAT3 signaling which can promote epithelial-mesenchymal transition (EMT), angiogenesis and immunosuppression in cancers (61). Han IH, et al. found that IL-6 induces M2 polarization and promotes proliferation of prostate cancer cells (62). And Zhang W, et al. showed that IL-6 promotes PD-L1 expression in monocytes and macrophages through JAK2/STAT1 and JAK2/STAT3/c-MYC signaling and induces immunosuppression in an orthotopic tumor transplantation model (63). Activated STAT3 by IL-6 also promotes the secretion of IL-10 and maintain the immunosuppressive function of Tregs (64).

#### 3.5.4 IL-10

IL-10 is a immunosuppressive cytokines secreted by immune cells, such as monocytes, macrophages and B cells. IL‐10 induces the TAM M2 polarization that further secrete high IL‐10, IL‐6, TGF‐β, which can promoting fibrosis and enhance tumor growth (65, 66). Patients with high level expression of IL-10 in both the serum and peritoneal effusions are correlated with advanced stage disease (67). By contact with its receptor, IL-10 can also activate the IL-10/STAT3 signaling pathway which skew macrophages to TAM M2 and promote high expression of various antiapoptosis, pro-tumorigenic and immunosuppression related genes (68). IL-10 also through TLR4/IL-10 signaling pathway alter macrophages to TAM M2 to promote epithelial-mesenchymal transition in pancreatic cancer cells (69). IL-10 expressed by TAMs suppresses IL-12 production by DCs, thus limit cytotoxic CD8+ T cell responses and resist chemotherapy. It could improve chemotherapy by blocking IL-10 receptor to enhance primary tumor response in breast cancer with paclitaxel and carboplatin treatment (70). It was also found that macrophages exposed to tumor culture supernatants secreting more IL-10 that may trigger a rise of the intratumoral forkhead/winged helix scurfy (FoxP3)^+^ Tregs population, which are associated with HCC aggressive (71).

#### 3.5.5 TNF

TNF mainly positively regulates M1 polarization by activating tumor necrosis factor receptor (TNFR) and the NF-kB signaling pathway to suppress M2 polarization. Other cytokines, such as myeloid differentiation primary response 88 (MyD88), can also inhibit M2 gene expression in TAMs, leading to an M1 phenotype (8, 72).

#### 3.5.6 TGF-β

TGF-β is a growth regulatory protein that shows both antitumoral and pro-tumoral activities. In the precancerous state, TGF-β inhibits cell proliferation, whereas in the established tumor stage, TGF-β enhances macrophage secretion of IL−10, which promotes macrophage polarization and induces immune evasion and metastasis. TGF−β secreted by TAMs promotes macrophage alteration to the pro−tumor M2 type (73).

## 4 Cancer immunotherapy of macrophages

Macrophages are trapped in the TME and promote the development and progression of tumors. Inflammation and cell death result in recruitment and maturation of macrophages into M1 TAMs. Hypoxia that enhances HIF genetic expression promotesre polarization of M2-type macrophages (9). The mode of tumors is considered ‘wounds never heal’, and the non-resolving tumor inflammatory response continues to recruit macrophages and mature them into the M1 phenotype, and with tumor development, hypoxia and anti-inflammatory cytokines transform macrophages to the M2 phenotype to promote tumor growth (9). Depending on the mode, improving tumor therapy should therefore consider blocking inflammation and blocking macrophage recruitment and eliminating preexisting TAMs (74). Because of diversity and heterogeneity of tumors, here, we use solid tumor therapeutic strategies as paradigm to explain as following (**Table 1**).

**Table 1:** Clinical trials of solid tumors associate of macrophage-targeting compounds.

Action	Compounds	Clinical phase	Tumor type and combination agent	Clinical trials
Elimination and blocking recruitment of M1 and M2 TAMs	Carlumab (CNTO888,CCL2 inhibitor)	I	Solid tumors + doxorubicin liposome injection; +gemcitabine;+ Paclitaxel and carboplatin;+ docetaxel	NCT01204996
	plerixafora (AMD-3100, CXCR4 antagonist)	II	Solid tumors	NCT01225419
	LY2510924 (CXCR4 antagonist peptide)	I	Solid tumors	NCT02737072
	Emactuzumab (RG7155, CSF-1R antibody)	I	Advanced Solid Tumors + RO5509554	NCT01494688
	Emactuzumab (RG7155, CSF-1R antibody)	I	Advanced solid tumors + Atezolizumab	NCT02323191
	PLX3397(Plexxikon, CSF-1R inhibitor),	Ib/II	Advanced solid tumors + paclitaxel	NCT01596751
	ARRY-382(CSF-1R inhibitor)	II	Advanced solid tumors + pembrolizumab	NCT02880371
	Pexidartinib (CSF-1R inhibitor)	I	Advanced solid tumors	NCT02734433
	BLZ945(CSF-1R inhibitor)	I	Advanced solid tumors	NCT02829723
	JNJ-40346527(CSF-1R inhibitor)	I	Prostate cancer	NCT03177460
	IMC-CS4(CSF-1R inhibitor)	I	Advanced solid tumors	NCT01346358
	FPA008 (Cabiralizumab, CSF-1R antibody)	I	Advanced solid tumors + nivolumab	NCT02526017
	PXL7486(CSF-1R inhibitor)	I	Advanced solid tumors	NCT01804530
	AMG820 (Amgen, CSF-1R inhibitor)	I	Solid tumors	NCT01444404
	SNFX-6352 (CSF-1R antagonists)	I	Advanced solid tumors + Durvalumab	NCT03238027
	Trabectedin	I	Solid tumor, Adult + Durvalumab	NCT03496519
Reprogramming TAM M2 to M1 to antitumor	Hu5F9-G4(CD47-SIRPα inhibitor)	I	Advanced solid malignancies	NCT02216409
	Hu5F9-G4(CD47-SIRPα inhibitor)	I	Advanced solid malignancies and colorectal carcinoma + cetuximab	NCT02953782
	TTI-621 (SIRPα-IgG1 Fc)	I	Solid tumors + Rituximab or Nivolumab	NCT02663518
	Selicrelumab(CD40 agonist)	I	Solid tumors + Atezolizumab	NCT02304393
	Selicrelumab(CD40 agonist)	I	Advanced solid tumors + Vanucizumab or Bevacizumab	NCT02665416
	SEA-CD40(CD40 agonist)	I	Solid tumors + pembrolizumab	NCT02376699
	IMO-2125(TLR9 agonist)	I	Refractory solid tumors, metastatic melanoma	NCT03052205
	SD101(TLR9 agonist)	I/II	Solid tumors + SBRT + pembrolizumab	NCT03007732
	Anakinra (IL-1R antagonist)	I	Advanced solid tumors + everolimus	NCT01624766
	GSK1795091(TLR4 agonist)	I	Advanced solid tumors + GSK3174998 anti- OX40) or (GSK3359609 anti- ICOS) or pembrolizumab	NCT03447314
	Telratolimod (MEDI91973, TLR7/8 agonist)	I	Solid tumors + Durvalumab and/or Palliative Radiation	NCT02556463
	IPI-549(PI3Kγ inhibitor)	Ib	Advanced solid tumors + nivolumab	NCT02637531
	Vanucizumab(Vasculature-modulating agent Ang2/VEGF)	I	Advanced/metastatic solid tumors	NCT02665416
	EF-022(Efranat, vitamin-D-binding protein, macrophage-activating factor)	I	Solid tumors	NCT02052492

Data were obtained from http://clinicaltrials.gov.

CCL2, C-C motif chemokine ligand 2; CCL5, C-C motif chemokine ligand 5; CCR2, C-C motif chemokine receptor 2; CXCR2, C-X-C chemokine receptor type 2;CSF-1,colony-stimulating factor-1; CSF-1R, Colony stimulating factor 1 receptor; CARMs, chimeric antigen receptor macrophages; EMT, epithelial mesenchymal transition; Fizz1, found in inflammatory zone 1; GM-CSF, granulocyte-macrophage colony-stimulating factor; HIF, hypoxia-inducible factors; HCC, hepatocellular carcinoma; HMGB-1, high mobility group box 1; IFN-γ, interferon-γ; IL-6, interleukin-6; IMCs, immature myeloid cells; LPS, lipopolysaccharide; M-CSF, macrophage colony-stimulating factor; MDSCs, myeloid-derived suppressor cells; MMP9, matrix metalloprotease 9; MyD88, myeloid differentiation primary response 88; PD-L1: programmed cell death ligand 1; PD-1, programmed cell death protein 1; PDGF, platelet-derived growth factor; RP-182, a synthetic 10-mer amphipathic analog of host defense peptides; ROS, reactive oxygen species; SREBP1,sterol regulatory element binding protein 1; SIRPa, signal regulatory protein alpha;TAMs, tumor−associated macrophages; TME,tumor microenvironment; TNF, tumor necrosis factor; TNFR, tumor necrosis factor receptor; TGF-β: transforming growth factor beta; VEGF, vascular endothelial growth factor.

### 4.1 blocking CCL2-CCR2 and CXCR4-CXCL12 signaling

Blocking the CCL2-CCR2 axis and CXCR4‐CXCL12 signaling pathway can prevent TAM recruitment and infiltration into the TME (74), which has shown potential therapeutic value for solid tumors in preclinical and clinical studies (**Table 1**). For example, an anti-CCL2 antibody, carlumab (CNTO888), can inhibitor macrophage infiltration to the tumor in mice, which has been applied in clinical trials to treat solid tumors and metastatic castrate-resistant prostate cancer (75). Clinical studies indicated that single-agent carlumab only temporarily repressed serum CCL2, resulting in no significant antitumor effects (76). However, combination of carlumab with several conventional chemotherapy regimens such as paclitaxel and carboplatin, significantly enhance the antitumor response (77)

Also, inhibition CXCR4‐CXCL12 signaling can more specifically promote TAM exclusion. CXCL12 is a cancer-associated fibroblast derived factor which recruit CXCR4-expressing monocytes toTME and skew to M2-like macrophages to promote tumor growth (78). It was found that targeting the CXCR4-CXCL12 signaling could effective treat for solid tumors in the clinic trails. For example, a CXCR4 antagonist Plerixafor (AMD3100), which can inhibit the secretion of VEGF-A from TAMs and lead to reduce tumor angiogenesis, has been used in clinical trials for treating solid tumors and children cancer (73). Other CXCR4 antagonist, such as LY2510924 (CXCR4 antagonist peptide) also use in clinical trials for treating solid tumors (74, 79).

### 4.2 blocking CSF-1/CSF-1R signaling

As mention before, the CSF-1/CSF-1R signaling pathway also plays a key role in TAM recruitment and polarization. Therefore, blocking the signal in TAMs has been developed in clinical trials for solid tumor therapy (80). For example, a monoclonal antibody Emactuzumab (RG7155) could effectively inhibit CSF-1R activation. Emactuzumab treatment significantly reduces CSF-1R+/CD163+ macrophages in diffuse-type giant cell tumor and increases the ratio of CD8+/CD4+. Emactuzumab in combination with chemotherapy and immunotherapy are underway in clinical trials of solid tumor treatment (74). CSF-1R specific inhibitors, such as PLX3397, PXL7486, AMG820, BLZ945, et al., also have been used in clinical trials for treatment of solid tumor. It was found that both CSF-1R antibodies and inhibitors could improve therapy in preclinical and clinical trial. For example, the CSF-1R inhibitor BLZ945, alone or in combination with anti-PD1 antibody immunotherapy could block macrophage recruitment and alter macrophage polarization to antitumor type that currently was being assessed in clinical trials for advanced-stage solid tumors treatment (74).

### 4.3 blocking CD47-SIRPα signaling

Although eliminating and inhibiting recruitment TAM strategies can delay tumor progression, these therapeutic approaches may have systemic toxicities as they target all macrophages without specific, and eliminating TAMs can be rapid compensation by tumor-associated neutrophils (TANs). It was found that withdrawal of CCL2/CCR2 inhibitors might accelerate metastasis in breast cancer by dramatically releasing of monocytes which were trapped in the bone marrow (81). So, it is appealing new strategies such as re-educating macrophages to anti-tumor phenotypes to overcome these limitations.

One method of re-educating macrophages is using inhibitors to block receptor signals on macrophages that modulate phagocytosis. Tumor cells overexpress the “don’t eat me” signaling molecule CD47, which suppresses macrophage phagocytic capacity by interacting with signal regulatory protein alpha (SIRPa). Using anti-CD47 antibodies to disrupt the CD47-SIRPα axis can restore the ability of macrophages to engulf tumors (74). Many conventional anti-CD47 antibodies have been demonstrated to be successful in preclinical and clinical trials. For example, it was found Hu5F9-G4, an anti-CD47 antibody, could inhibit the interaction of CD47 with SIRPα and promoted macrophage-mediated phagocytosis to kill cancer cells. Hu5F9-G4 has been used in clinical trials to treat solid tumors and various hematological malignancies (82).

Also, the polypeptides or recombinant proteins including engineered high-affinity SIRPα protein which derived from SIRPα can act as decoy bind to CD47 to disrupt the CD47-SIRPα signaling. Studies showed that recombinant protein TTI-621 which composed of the N-terminal domain of SIRPα fused to human IgG1 could suppress tumor growth by increasing macrophage-mediated phagocytosis of solid tumor cells (83). TTI-621 is now in clinical investigation to treat solid tumors.

### 4.4 CD40 agonists

CD40 is a superfamily member of TNF receptor and expresse on many antigen-presenting cells (APCs) as well as some tumor cells. It was found that agonistic anti-CD40 antibodies could stimulate TAMs to promote the secretion of the proinflammatory cytokines such as NO and TNF-α to activate effector T cells to reestablish tumor immune surveillance. It was found that many agonistic anti-CD40 antibodies such as Selicrelumab (CD40 agonist) in combination with immunotherapy significantly promoted macrophages phagocytic activity to antitumor. Selicrelumab combination with immunotherapy such as atezolizumab has been use in clinical trials to treat solid tumors (74).

### 4.5 Toll-like receptor agonist

Toll-like receptors (TLRs) play critical roles in activating the innate immune reaction of macrophages toward antitumor M1 phenotype. Activation of multiple TLR signals promotes phagocytic activity of macrophages and enhances antitumor responses. For example, it was found that TLR4 and TLR5 agonists could polarize more CD206+ M2 TAMs to CD86+ M1 phenotype and suppressed tumor growth without obvious toxicity. Other TLR agonists have also been found to alter f M2 TAMs to pro-inflammatory M1 phenotype and promote tumor regression in mouse models (84). TLR9 agonist IMO-2125, which can induce tumor regression by promoting macrophage polarization to antitumor type, has been evaluated in clinical trials to treat refractory solid tumors and metastatic melanoma (74). However, TLR stimulation by agonist always lead to PD-L1 expressed level elevation in macrophages, resulting in limiting antitumor responds. To overcome this setback, IMO-2125 combined with immunotherapy such as iplimumab to treat cancer more effective. Recently, MO-2125 combined with iplimumab was approved by FDA to treatment of melanoma. Others TLR9 agonist, such as SD101 was also investigation along with PD-1 blockade in clinical trials to enhance therapeutic efficacy (74, 85).

### 4.6 PI3Kγ inhibitor and other treatments promoting macrophage reprogramming

Phosphatidylinositide 3-kinases (PI3K), which can specifically phosphorylate the 3′ position in the inositol moiety of phospholipids, play crucial roles in inflammatory, immunosuppression associated with cancer or autoimmune diseases. PI3Kγ is the class IB PI3K member which playing significant roles in immunosuppressive transcriptional programming by contacting with G protein (86). PI3Kγ promotes transcription of genes and enhance immunosuppressive factors Arg1, TGF-β, and IL‐10 expression that links to the M2 immunosuppressive macrophage phenotype (87).

PI3Kγ inhibitors can alter macrophages toward proinflammatory phenotype and block recruitment of macrophages and neutrophils from peripheral blood (88, 89). IPI‐549, the PI3Kγ‐selective inhibitor, has been reported to promote macrophage polarization to M1 states and enhancing immunotherapy by increasing CD8+ T‐cell activation and cytotoxicity (90). IPI-549 combination with nivolumab has been investigated in phase I clinical trials for several advanced solid tumors.

Other classical treatments such as blocking the function of TAM-expressed PD-L1to promote macrophage reprogramming to enhance antitumor effects. TAMs expressing the checkpoint molecule PD-L1 negatively regulate the phagocytic ability of TAMs and suppress cytotoxic T-cell immunity against tumor cells. Blocking the PD-1/PD-L1 pathway can enhance the phagocytosis of macrophages and prolong the survival of mice in cancer models (91). It also improved therapy in clinical treatment by blocking the function of TAM-expressed PD-L1 (92).

Recently, a new technique of re-educating macrophages to generate chimeric antigen receptor macrophages (CARMs) has emerged for cell-based cancer immunotherapy. It was found that CARMs encoding the CD3ζ intracellular domain can target the tumor antigen mesothelin or HER2 and kill antigen-positive solid tumor cells (93, 94). A huge breakthrough was shown in CARM immunotherapy on July 27, 2020, and the FDA approved the investigational new drug application for anti-human HER2-CARM (CT-0508) to treat recurrent or metastatic HER2-overexpressing solid tumors (79).

## 5 Conclusions and perspectives

Inflammation is a double-edged sword in tumor treatment. It should distinguish ‘antitumor inflammation (acute inflammation)’ and ‘pro-tumor inflammation (chronic inflammation)’ for precision tumor therapy. ‘Antitumor inflammation’ can active the immune system that recognize and cause tumor cell death by immune surveillance process. But chronic inflammation promotes immunosuppression and tumor progression (95).TAMs polarize and orchestrate tumor-related inflammation in TME. M1 phenotype secrete pro-inflammatory cytokines (TNF-α, IL-1β, IL-12, e.g.), and co-stimulatory molecules to present antigen efficiently and promote Th1 response to destroy tumor cells. However, M2 TAM secrete anti-inflammatory and immunosuppressive molecules (IL-4, IL-10, TGF-β, e.g.), to promote chronic inflammation that lead to sustained recruit and polarize TAMs to the TME and promote tumor malignant transformation.

Targeting TAM therapeutic protocols, such as eliminating and inhibiting recruitment, switching the M2 phenotype to the M1 phenotype, enhancing phagocytosis and increasing antigen presentation to kill tumor cells, and new CARM technology have also greatly improved cancer treatment. However, these cancer treatment technologies are still a long way off. The biggest difficulty is how to precisely promote the ‘antitumor inflammation’ inducing by macrophage to kill tumor cells and eliminate pro-tumor chronic inflammation in tumor therapy. For example, eliminating and inhibiting recruitment TAM strategies to treat inflammatory tumor may have systemic toxicities as they target all macrophages including M1and tissue-resident macrophages without specific that will leads to increased bacterial infections, metastasis and accelerated death. And switching the M2 to the M1 phenotype may only result in temporary and limited antitumor efficacy. Because of tumor heterogeneity, it is difficult to definite TAMs subpopulations in different human tumors. Also, TAMs are not stably inherited and they can change in TME. By contact with tumor cells, M1may sustained polarize to M2 to promote malignancy progression. In addition, although anti-tumor inflammation producing by TAM M1 can cause tumor cell death, it can also create a mutagenic microenvironment which may lead to TAM polarization to M2 resulting in promoting tumor progress.

So, it need to explore new strategies that not only renovate the inflammatory tumor “soil” that consist by the TAMs to construct a anti-tumor microenvironment, but also kill the tumor “seeds” in the “soil”. Thus, continuous studies are needed to elucidate the mechanisms that drive phenotypic changes in TAMs in the inflammatory TME, which will help in the development of cancer immunotherapy of macrophages.

## Author contributions

BL, ZZ gathered the related literature, prepared the figures and drafted the manuscript. HL and ML participated in the design of the review and drafted the manuscript. All authors read and approved the final manuscript.
